# Supplementary material for: Frontal cortex hyperactivation and gamma desynchrony in Fragile X syndrome: Correlates of auditory hypersensitivity
Source: PLoS One. 2025 May 20;20(5):e0306157. doi: 10.1371/journal.pone.0306157 (PMC12091838; doi:10.1371/journal.pone.0306157)
Supplement: S1 Table — Includes MNI coordinates and vertex counts. (DOCX) [file pone.0306157.s001.docx]

**Supplementary Table 1. Assignment of cortical nodes to region groupings as attributed by the Desikan-Killiany (DK) atlas.**

|  |  |  | MNI Centroid | | |  |
| --- | --- | --- | --- | --- | --- | --- |
| Node | Abbreviation | Cortex | x | y | z | Vert. |
| Caudal Anterior Cingulate R | cACC R | RL | 4 | 22 | 27 | 68 |
| Isthmus Cingulate L | iCC L | LL | -7 | -45 | 18 | 81 |
| Isthmus Cingulate R | iCC R | RL | 6 | -42 | 19 | 91 |
| Lateral Orbitofrontal L | LOF L | LPF | -25 | 30 | -19 | 221 |
| Lateral Orbitofrontal R | LOF R | RPF | 23 | 32 | -19 | 206 |
| Medial Orbitofrontal L | MOF L | LPF | -7 | 33 | -17 | 155 |
| Medial Orbitofrontal R | MOF R | RPF | 4 | 37 | -15 | 170 |
| Parahippocampal L | paraH L | LT | -26 | -29 | -21 | 68 |
| Parahippocampal R | paraH R | RT | 24 | -30 | -19 | 66 |
| Posterior Cingulate L | PCC L | LL | -5 | -15 | 38 | 85 |
| Posterior Cingulate R | PCC R | RL | 5 | -17 | 39 | 93 |
| Precuneus L | PCUN L | LP | -9 | -59 | 38 | 314 |
| Precuneus R | PCUN R | RP | 10 | -58 | 38 | 325 |
| Rostral Anterior Cingulate L | rACC L | LL | -5 | 39 | 1 | 78 |
| Rostral Anterior Cingulate R | rACC R | RL | 4 | 38 | 3 | 56 |
| Caudal Middle Frontal L | cMFG L | LF | -37 | 11 | 47 | 224 |
| Caudal Middle Frontal R | cMFG R | RF | 37 | 13 | 48 | 186 |
| Insula L | INS L | LT | -38 | -2 | 2 | 174 |
| Insula R | INS R | RT | 36 | 2 | -2 | 196 |
| Rostral Middle Frontal L | rMFG L | LF | -34 | 47 | 17 | 543 |
| Rostral Middle Frontal R | rMFG R | RF | 34 | 48 | 17 | 551 |
| Supramarginal L | SMAR L | LP | -57 | -38 | 34 | 305 |
| Supramarginal R | SMAR R | RP | 54 | -31 | 36 | 302 |
| Caudal Anterior Cingulate L | cACC L | LL | -5 | 21 | 26 | 48 |
| Inferior Temporal L | ITG L | LT | -53 | -36 | -22 | 307 |
| Inferior Temporal R | ITG R | RT | 51 | -32 | -25 | 316 |
| Middle Temporal L | MTG L | LT | -58 | -23 | -15 | 277 |
| Middle Temporal R | MTG R | RT | 58 | -22 | -15 | 324 |
| Pars Opercularis L | pOPER L | LF | -49 | 17 | 14 | 139 |
| Pars Opercularis R | pOPER R | RF | 49 | 17 | 14 | 118 |
| Pars Orbitalis L | pORB L | LPF | -44 | 39 | -14 | 72 |
| Pars Orbitalis R | pORB R | RPF | 43 | 42 | -15 | 68 |
| Pars Triangularis L | pTRI L | LF | -47 | 32 | 1 | 101 |
| Pars Triangularis R | pTRI R | RF | 48 | 34 | 2 | 148 |
| Superior Temporal L | STG L | LT | -55 | -12 | -4 | 290 |
| Superior Temporal R | STG R | RT | 54 | -6 | -7 | 257 |
| Cuneus L | CUN L | LO | -6 | -80 | 19 | 93 |
| Cuneus R | CUN R | RO | 8 | -78 | 20 | 99 |
| Fusiform L | FUS L | LT | -36 | -43 | -22 | 268 |
| Fusiform R | FUS R | RT | 35 | -41 | -23 | 255 |
| Lateral Occipital L | LOG L | LO | -31 | -89 | 0 | 371 |
| Lateral Occipital R | LOG R | RO | 35 | -85 | 2 | 367 |
| Lingula L | LING L | LO | -14 | -71 | -5 | 246 |
| Lingula R | LING R | RO | 13 | -67 | -4 | 227 |
| Banks of Sup. Temp. Sulcus L | BSTS L | LT | -53 | -45 | 8 | 76 |
| Banks of Sup. Temp. Sulcus R | BSTS R | RT | 54 | -41 | 10 | 70 |
| Entorhinal L | ENT L | LT | -26 | -5 | -33 | 30 |
| Entorhinal R | ENT R | RT | 23 | -6 | -35 | 32 |
| Frontal Pole L | FP L | LPF | -7 | 68 | -11 | 22 |
| Frontal Pole R | FP R | RPF | 7 | 68 | -15 | 30 |
| Inferior Parietal L | IPL L | LP | -42 | -71 | 32 | 351 |
| Inferior Parietal R | IPL R | RP | 46 | -63 | 32 | 421 |
| Paracentral L | paraC L | LC | -7 | -30 | 57 | 110 |
| Paracentral R | paraC R | RC | 7 | -27 | 57 | 128 |
| Pericalcarine L | periCAL L | LO | -11 | -82 | 6 | 109 |
| Pericalcarine R | periCAL R | RO | 12 | -80 | 7 | 110 |
| Postcentral L | postC L | LC | -46 | -22 | 45 | 333 |
| Postcentral R | postC R | RC | 44 | -20 | 46 | 307 |
| Precentral L | preC L | LC | -41 | -9 | 46 | 339 |
| Precentral R | preC R | RC | 40 | -7 | 46 | 353 |
| Superior Frontal L | sFG L | LF | -12 | 30 | 41 | 671 |
| Superior Frontal R | sFG R | RF | 12 | 32 | 41 | 603 |
| Superior Parietal L | SPL L | LP | -23 | -65 | 50 | 484 |
| Superior Parietal R | SPL R | RP | 24 | -65 | 51 | 464 |
| Temporal Pole L | TP L | LT | -28 | 14 | -38 | 38 |
| Temporal Pole R | TP R | RT | 27 | 16 | -36 | 38 |
| Transverse Temporal L | TT L | LT | -46 | -23 | 10 | 34 |
| Transverse Temporal R | TT R | RT | 46 | -17 | 9 | 23 |

The MNI coordinates and number of vertices included in each node parcel are also displayed. We considered nodes as replicates within regions within our linear models. Abbreviations: L, Left; R, right; F, frontal; L, cingulate; O, occipital; P, parietal; PF, prefrontal; T, temporal; MNI, Montreal Neurologic Institute; Vert., Number of Vertices.
