# Supplementary material for: Frontal cortex hyperactivation and gamma desynchrony in Fragile X syndrome: Correlates of auditory hypersensitivity
Source: PLoS One. 2025 May 20;20(5):e0306157. doi: 10.1371/journal.pone.0306157 (PMC12091838; doi:10.1371/journal.pone.0306157)
Supplement: S2 Table — (DOCX) [file pone.0306157.s002.docx]

**Supplementary Table 2: Summary of linear mixed model results.**

| Model | Predictor | numDF | denDF | F-value | p-value |
| --- | --- | --- | --- | --- | --- |
| **ITC: 40 Hz** | (Intercept) | 1 | 4,973 | 16987.5 | <.001 |
|  | group | 1 | 71 | 6.9 | .011 |
|  | sex | 1 | 71 | 3e-04 | .986 |
|  | region | 13 | 4,973 | 38.9 | <.001 |
|  | group:sex | 1 | 71 | 3.9 | .053 |
|  | group:region | 13 | 4,973 | 1.5 | .124 |
|  | sex:region | 13 | 4,973 | 1.7 | .058 |
|  | group:sex:region | 13 | 4,973 | 1.9 | .025 |
| **ITC: 80 Hz** | (Intercept) | 1 | 4,973 | 16135.3 | <.001 |
|  | group | 1 | 71 | 3.9 | .053 |
|  | sex | 1 | 71 | .8 | .367 |
|  | region | 13 | 4,973 | 15.2 | <.001 |
|  | group:sex | 1 | 71 | .6 | .456 |
|  | group:region | 13 | 4,973 | 4.6 | <.001 |
|  | sex:region | 13 | 4,973 | 1.5 | .121 |
|  | group:sex:region | 13 | 4,973 | 1.2 | .242 |
| **ITC: Onset** | (Intercept) | 1 | 4,973 | 7853.2 | <.001 |
|  | group | 1 | 71 | 4.3 | .042 |
|  | sex | 1 | 71 | .01 | .936 |
|  | region | 13 | 4,973 | 51.9 | <.001 |
|  | group:sex | 1 | 71 | .1 | .728 |
|  | group:region | 13 | 4,973 | 2.9 | <.001 |
|  | sex:region | 13 | 4,973 | 2.8 | <.001 |
|  | group:sex:region | 13 | 4,973 | 1.4 | .142 |
| **ITC: Offset** | (Intercept) | 1 | 4,973 | 14939.9 | <.001 |
|  | group | 1 | 71 | 1.5 | .218 |
|  | sex | 1 | 71 | .01 | .922 |
|  | region | 13 | 4,973 | 21.8 | <.001 |
|  | group:sex | 1 | 71 | .1 | .783 |
|  | group:region | 13 | 4,973 | 1.7 | .062 |
|  | sex:region | 13 | 4,973 | .9 | .568 |
|  | group:sex:region | 13 | 4,973 | 1.7 | .047 |
| **STP: Gamma1** | (Intercept) | 1 | 4,973 | 1530204.9 | <.001 |
|  | group | 1 | 71 | 12.7 | <.001 |
|  | sex | 1 | 71 | 36.4 | <.001 |
|  | region | 13 | 4,973 | 434.0 | <.001 |
|  | group:sex | 1 | 71 | 4.2 | .045 |
|  | group:region | 13 | 4,973 | 2.6 | .002 |
|  | sex:region | 13 | 4,973 | 2.6 | .001 |
|  | group:sex:region | 13 | 4,973 | .7 | .799 |
| **STP: Gamm2** | (Intercept) | 1 | 4,973 | 971679.6 | <.001 |
|  | group | 1 | 71 | 1.4 | .235 |
|  | sex | 1 | 71 | 13.7 | <.001 |
|  | region | 13 | 4,973 | 461.9 | <.001 |
|  | group:sex | 1 | 71 | 7.8 | .007 |
|  | group:region | 13 | 4,973 | 2.5 | .002 |
|  | sex:region | 13 | 4,973 | 2.3 | .006 |
|  | group:sex:region | 13 | 4,973 | .5 | .943 |
| **STP: Alpha** | (Intercept) | 1 | 4,973 | 684410.9 | <.001 |
|  | group | 1 | 71 | 4.0 | .048 |
|  | sex | 1 | 71 | 1.7 | .190 |
|  | region | 13 | 4,973 | 171.5 | <.001 |
|  | group:sex | 1 | 71 | 6.4 | .014 |
|  | group:region | 13 | 4,973 | 2.8 | <.001 |
|  | sex:region | 13 | 4,973 | 5.5 | <.001 |
|  | group:sex:region | 13 | 4,973 | 9.6 | <.001 |
| **ERSP: Gamma1** | (Intercept) | 1 | 4,973 | 8.2 | .004 |
|  | group | 1 | 71 | 2.4 | .123 |
|  | sex | 1 | 71 | 3.9 | .052 |
|  | region | 13 | 4,973 | 2.1 | .011 |
|  | group:sex | 1 | 71 | .03 | .866 |
|  | group:region | 13 | 4,973 | 1.5 | .122 |
|  | sex:region | 13 | 4,973 | .8 | .663 |
|  | group:sex:region | 13 | 4,973 | 2.7 | <.001 |
| **ERSP: Gamma2** | (Intercept) | 1 | 4,973 | 4.9 | .027 |
|  | group | 1 | 71 | 1.0 | .330 |
|  | sex | 1 | 71 | 3.7 | .059 |
|  | region | 13 | 4,973 | 2.5 | .002 |
|  | group:sex | 1 | 71 | .2 | .659 |
|  | group:region | 13 | 4,973 | 2.7 | <.001 |
|  | sex:region | 13 | 4,973 | 2.4 | .004 |
|  | group:sex:region | 13 | 4,973 | 2.0 | .017 |
| **ERSP: Alpha** | (Intercept) | 1 | 4,973 | 13.7 | <.001 |
|  | group | 1 | 71 | 1.3 | .251 |
|  | sex | 1 | 71 | 3.1 | .081 |
|  | region | 13 | 4,973 | 6.2 | <.001 |
|  | group:sex | 1 | 71 | 6.1 | .016 |
|  | group:region | 13 | 4,973 | 1.6 | .079 |
|  | sex:region | 13 | 4,973 | 1.0 | .403 |
|  | group:sex:region | 13 | 4,973 | 2.8 | <.001 |

A series of linear mixed-effects models (LME) were conducted for each of the ten power and phase response variables (see Methods). To account for individual variation in electrode position, we opted to perform statistical modeling at the region level and use nodes within a region as replicates. Fixed effects included Group (FXS or TDC), Sex (male or female), and Region (14).
