# Supplementary material for: Frontal cortex hyperactivation and gamma desynchrony in Fragile X syndrome: Correlates of auditory hypersensitivity
Source: PLoS One. 2025 May 20;20(5):e0306157. doi: 10.1371/journal.pone.0306157 (PMC12091838; doi:10.1371/journal.pone.0306157)
Supplement: S1 Fig — A. Amplitude-modulated pink noise carrier waveform. B. Waterfall plot of broadband power spectrum. (DOCX) [file pone.0306157.s003.docx]

**Supplementary Figure 1: Broadband auditory chirp stimulus.**


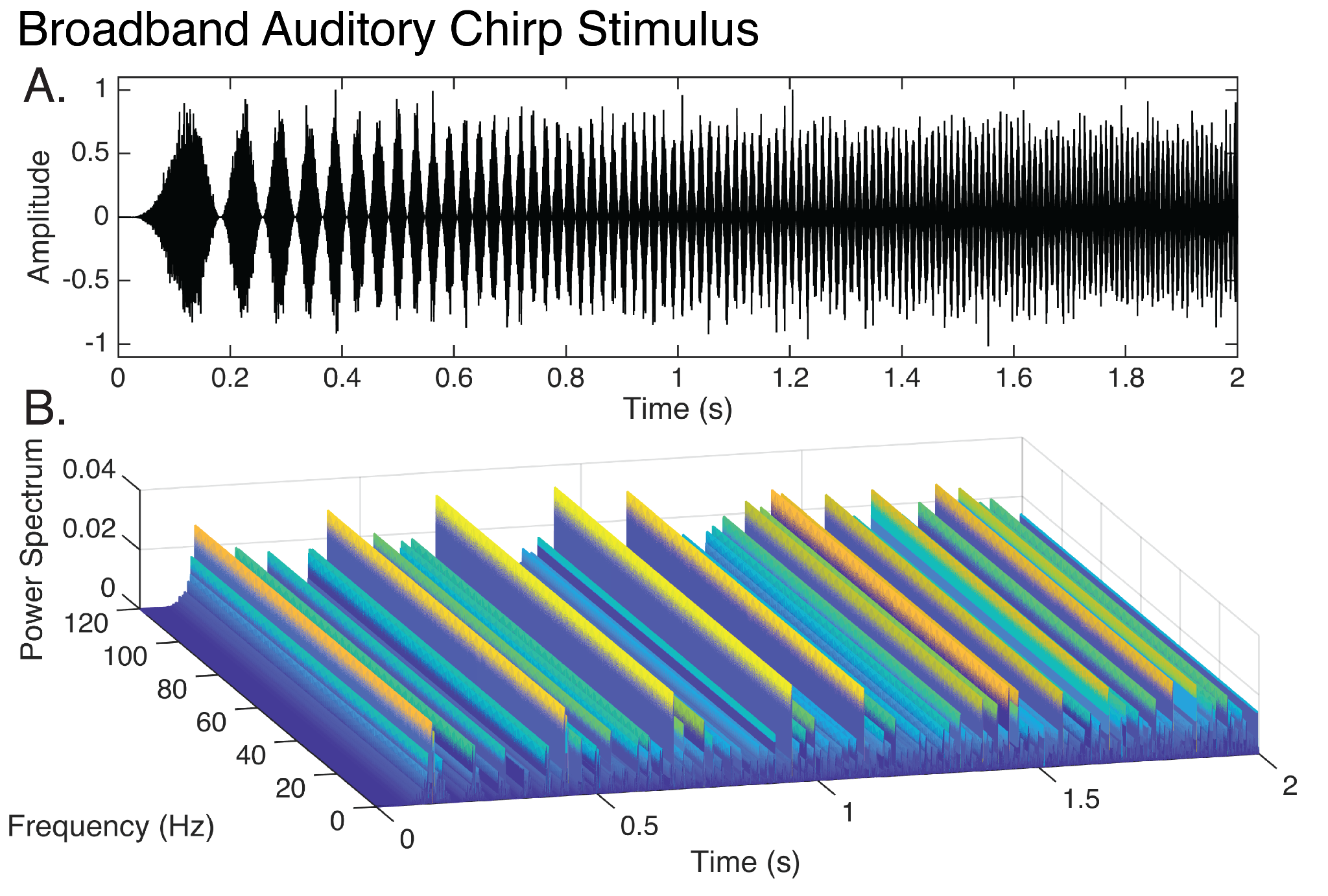


**Supplementary Figure 1:** A. Plot of amplitude-modulated pink noise carrier waveform. Frequency of modulation increases linearly from 0 to 120 Hz over 2 seconds. B. Waterfall plot depicting broadband power spectrum of broadband chirp stimulus.
