# Supplementary material for: Frontal cortex hyperactivation and gamma desynchrony in Fragile X syndrome: Correlates of auditory hypersensitivity
Source: PLoS One. 2025 May 20;20(5):e0306157. doi: 10.1371/journal.pone.0306157 (PMC12091838; doi:10.1371/journal.pone.0306157)
Supplement: S2 Fig — Depicts 14 hierarchical regions of the Desikan-Killiany atlas encompassing 68 cortical nodes. (DOCX) [file pone.0306157.s004.docx]

**Supplementary Figure 2: Atlas Regions**


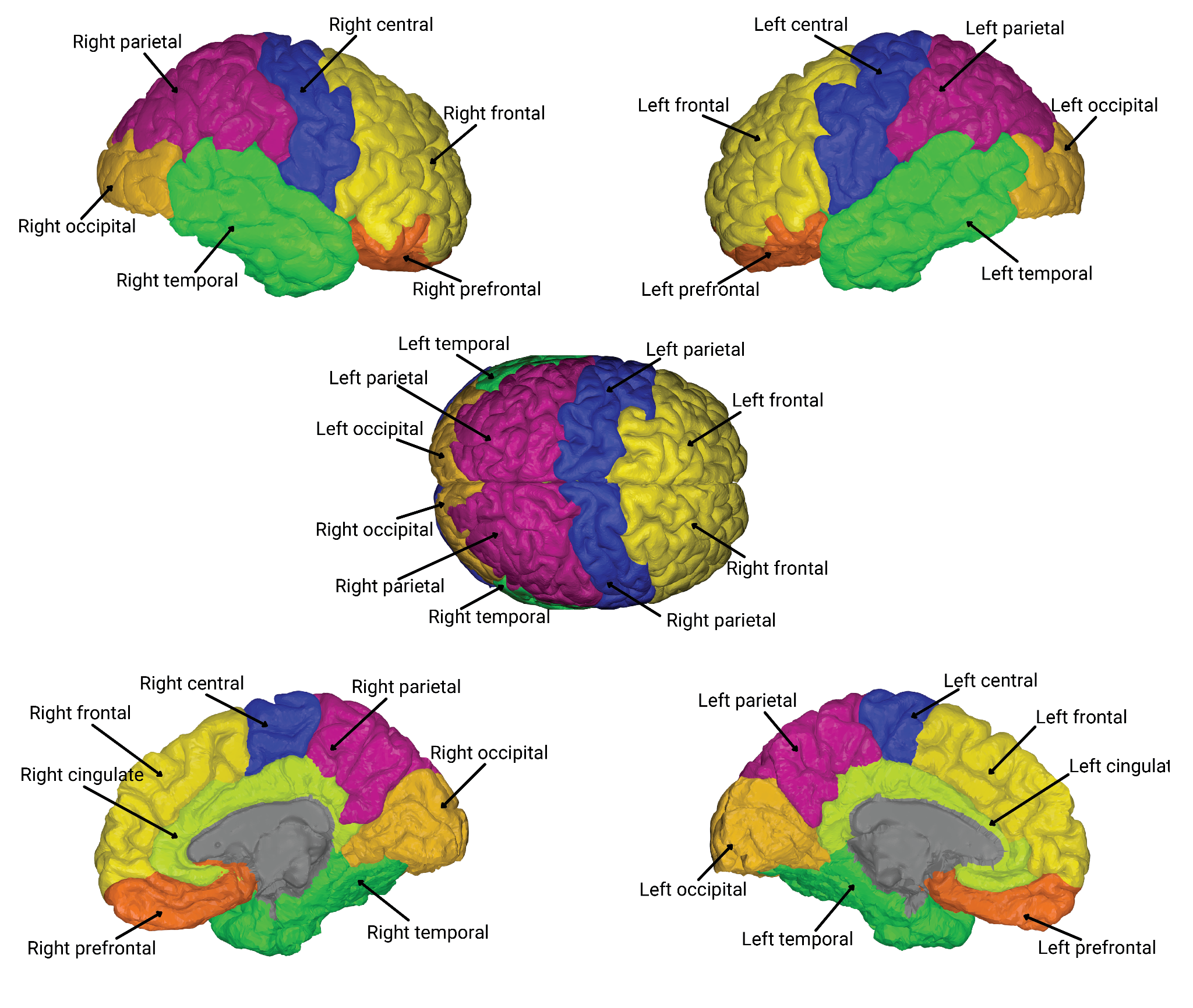


**Supplementary Figure 2:** The Desikan-Killiany atlas was used to group vertex parcellations following source localization. A total of 14 hierarchical regions (shown below) encompass 68 cortical nodes as described in Supplemental Table 1.
