# Supplementary material for: Frontal cortex hyperactivation and gamma desynchrony in Fragile X syndrome: Correlates of auditory hypersensitivity
Source: PLoS One. 2025 May 20;20(5):e0306157. doi: 10.1371/journal.pone.0306157 (PMC12091838; doi:10.1371/journal.pone.0306157)
Supplement: S3 Fig — Shows nodes used for Renyi transfer entropy between frontal and superior temporal gyrus regions. (DOCX) [file pone.0306157.s005.docx]

**Supplementary Figure 3: Frontotemporal atlas nodes for transfer entropy estimation**


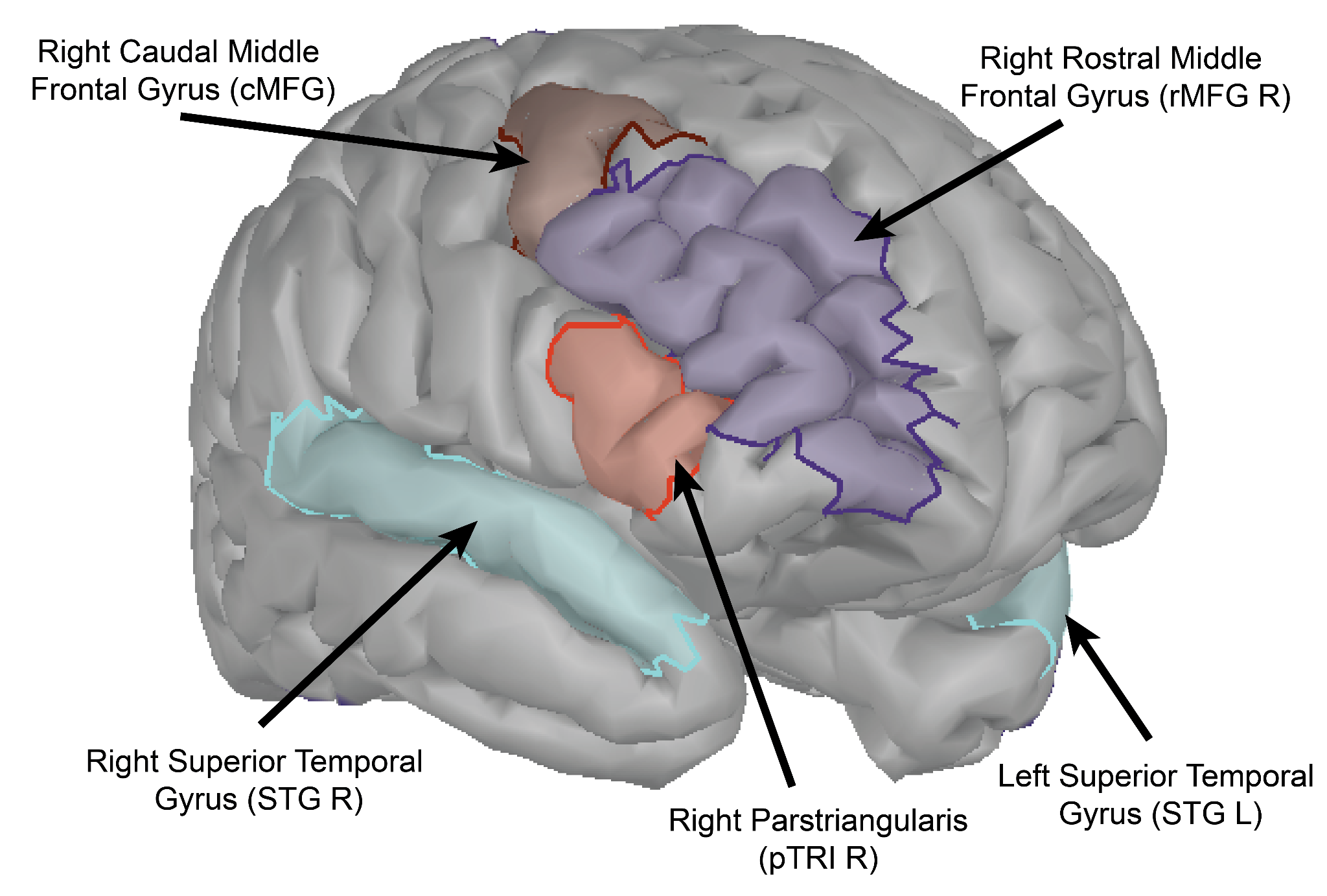


**Supplementary Figure 3:** Transfer entropy was calculated between frontotemporal nodes associated with significant group differences in onset and gamma ITPC. Onset ITPC was significantly increased within the caudal middle frontal R (cMFG; t=2.73, p=.001), pars triangularis R (pTRI, t =2.69, p=.011), and rostral middle frontal R (rMFG; t=2.44, p=.019) in FXS. Renyi transfer entropy was estimated between these frontal nodes and right and left superior temporal gyrus (STG), which is the node-level region that includes the auditory cortex.
